# Let’s get in sync: current standing and future of AI-based detection of patient-ventilator asynchrony

**DOI:** 10.1186/s40635-025-00746-8

**Published:** 2025-03-21

**Authors:** Thijs P. Rietveld, Björn J. P. van der Ster, Abraham Schoe, Henrik Endeman, Anton Balakirev, Daria Kozlova, Diederik A. M. P. J. Gommers, Annemijn H. Jonkman

**Affiliations:** 1 Adult Intensive Care, Erasmus Medical Center, Dr. Molewaterplein 40, Rotterdam, The Netherlands; 2 Intensive Care, Leiden University Medical Center, Leiden, The Netherlands; 3 Intensive Care, OLVG, Amsterdam, The Netherlands; 4 Deep Breath B.V, Rotterdam, The Netherlands

**Keywords:** Patient-ventilator asynchrony, Mechanical ventilation, ICU, Artificial intelligence, Rule-based algorithms, Machine learning, Deep learning, Bedside implementation

## Abstract

**Background:**

Patient-ventilator asynchrony (PVA) is a mismatch between the patient’s respiratory drive/effort and the ventilator breath delivery. It occurs frequently in mechanically ventilated patients and has been associated with adverse events and increased duration of ventilation. Identifying PVA through visual inspection of ventilator waveforms is highly challenging and time-consuming. Automated PVA detection using Artificial Intelligence (AI) has been increasingly studied, potentially offering real-time monitoring at the bedside. In this review, we discuss advances in automatic detection of PVA, focusing on developments of the last 15 years.

**Results:**

Nineteen studies were identified. Multiple forms of AI have been used for the automated detection of PVA, including rule-based algorithms, machine learning and deep learning. Three licensed algorithms are currently reported. Results of algorithms are generally promising (average reported sensitivity, specificity and accuracy of 0.80, 0.93 and 0.92, respectively), but most algorithms are only available offline, can detect a small subset of PVAs (focusing mostly on ineffective effort and double trigger asynchronies), or remain in the development or validation stage (84% (16/19 of the reviewed studies)). Moreover, only in 58% (11/19) of the studies a reference method for monitoring patient’s breathing effort was available. To move from bench to bedside implementation, data quality should be improved and algorithms that can detect multiple PVAs should be externally validated, incorporating measures for breathing effort as ground truth. Last, prospective integration and model testing/finetuning in different ICU settings is key.

**Conclusions:**

AI-based techniques for automated PVA detection are increasingly studied and show potential. For widespread implementation to succeed, several steps, including external validation and (near) real-time employment, should be considered. Then, automated PVA detection could aid in monitoring and mitigating PVAs, to eventually optimize personalized mechanical ventilation, improve clinical outcomes and reduce clinician’s workload.

**Supplementary Information:**

The online version contains supplementary material available at 10.1186/s40635-025-00746-8.

## Background

Patient-ventilator asynchrony (PVA) reflects a mismatch between the patient’s respiratory drive and the ventilator breath delivery [1–3] and is frequently observed in mechanically ventilated patients, especially during an assisted ventilation mode [4]. PVA has been associated with lung injury [5–7], diaphragm dysfunction [8, 9], sleep disruption [10, 11], longer duration of ventilation [12, 13] and higher mortality [13, 14].

PVA occurs in different forms [1, 15, 16] and is reported in up to 90% of mechanically ventilated patients with variable incidence and impact, but only short-time recordings and few PVA types were investigated [13, 17–19]. Identifying and resolving PVA through visual inspection of ventilator waveforms is challenging [1, 20, 21], and substantial disagreement between (expert) clinicians was reported [20].

Monitoring patient effort via esophageal pressure (Pes) or electrical activity of the diaphragm (EAdi) could facilitate and improve PVA detection, but is not widely available [3, 22, 23]. Furthermore, clinician’s time at the bedside is limited [24], while ventilators produce large amounts of complex, continuous data. This likely results in a vast underestimation of PVA prevalence and inaccurate classification of PVA type. Hence, the direct relationship with adverse physiological and clinical outcomes remains to be conclusively established [1, 15]. This strengthens the need for an automated and (near) real-time method of PVA detection.

Artificial intelligence (AI) has seen exponential improvements in the last decade and has been used for the automated detection of PVA [15, 25]. AI-based methods could allow future real-time bedside implementation and studying the pathophysiological impact of PVA throughout the full course of mechanical ventilation. This would build towards evidence-based and data-driven clinical recommendations on how to prevent and resolve PVA. In this review, we discuss advancements in the automatic detection of PVA, focusing on developments in the last 15 years. We grouped and evaluated algorithms based on their characteristics as either rule-based, machine learning (ML), deep learning (DL) or licensed software. Furthermore, we present common challenges and propose key steps that are needed for future implementation in clinical practice.

## Rule-based algorithms

Rule-based algorithms are one of the earliest, simplest forms of AI and include manually determined rules and thresholds [26], based on expert knowledge/consensus [27]. These algorithms have seen widespread application in clinical monitoring software [28], likely due to their transparent and comprehensible nature.

Rule-based algorithms have shown to be able to detect multiple types of PVAs, but their development and testing are currently limited to a small number of PVAs (Table 1). For example, Baedorf-Kassis et al. [29] focused on reverse triggering using pressure–volume loops (PV loops). They compared their rule-based algorithm to manual waveform and Pes annotation and found a sensitivity, specificity and F1-score of 0.75, 0.97 and 0.78, respectively. For interpretation purposes, we detail performance metrics that we mention throughout this paper in Supplemental File 1. Rodriguez et al. [30] classified reverse and double triggering (with and without breath stacking) using flow and airway pressure curves, but failed to validate detection of double triggering due to the low prevalence of this PVA in their cohort. They used a manual annotation of waveforms with and without Pes as reference standard. When Pes was included, they found a sensitivity of ≥ 0.86, specificity of ≥ 0.98 and overall accuracy of 0.92 for detecting reverse triggering. For the dataset without Pes, accuracy increased to 0.96, but sensitivity and specificity dropped to ≥ 0.74 and 0.80, respectively.

**Table 1:** Characteristics of selected rule-based algorithms (studies are arranged chronologically)

Author	Type of PVA	Population	Input data	Algorithm	Performance	Validation	Reference	Pes or EAdi	Comments
Rodriguez et al. [30]	Reverse trigger, double trigger	ARDS patients on volume-controlled ventilation	Paw and flow waveforms	Rule-based algorithm	*Pes validation set:* Accuracy: 0.92 Sensitivity ≥ 0.86 Specificity ≥ 0.91 *No Pes validation set:* Accuracy: 0.96 Sensitivity ≥ 0.74 Specificity ≥ 0.80	Pes validation set: 11 patients (710 breaths) No Pes validation: 99 patients (1881 breaths)	Two separate datasets; one with Pes available, one based on manual annotation of Paw and volume waveforms by 7 experts	Pes available in 1 validation set, no EAdi available	Not validated for double trigger since prevalence was too low
Baedorf-Kassis et al. [29]	Reverse trigger	15 ARDS patients (23,321 breaths)	Flow, pressure, volume and Pes waveforms, Campbell diagrams (PV loops)	Rule-based software and modified ResNet network	*Rule-based model:* F1-score: 0.78 Sensitivity: 0.75 Specificity: 0.97 *Deep NN:* F1-score: 0.77 Sensitivity: 0.73 Specificity: 0.97	13 Patients (9923 breaths)	Manual annotation of waveforms (flow, pressure, volume) and Pes by 2 experts	Pes available, no EAdi available	Neural network and an iteration of the rule-based algorithm used Pes in classification

*ARDS* Acute Respiratory Distress Syndrome, *EAdi* Electrical Activity of the Diaphragm, *NN* Neural Network, *Paw* Airway Pressure, *Pes* Esophageal Pressure, *PV loops* Pressure-Volume loops, *PVA* Patient-Ventilator Asynchrony

## Machine learning (ML)

ML is a sub-field of AI characterized by its ability to learn from input data [26]. ML methods are based on more complex statistical calculations, resulting in a less transparent architecture than rule-based algorithms, but often perform better on complex data. Various ML methods for PVA detection have been proposed (Table 2). For example, Gholami et al. [31] used a random forest model for the detection of premature and delayed cycling based on several ‘features’ per breath from the pressure-, flow- and derived delta waveform (the difference between the normalized pressure and flow). As ground truth, they performed manual annotation of flow, pressure and volume recordings, but no Pes measurement was available. For premature cycling, a sensitivity and specificity of 0.89 and 0.99 was reported, while for delayed cycling, a sensitivity and specificity of 0.94 and 0.98 was achieved.

**Table 2:** Characteristics of selected machine learning algorithms (studies are arranged chronologically)

Author	Type of PVA	Population	Input data	Algorithm	Performance	Validation	Reference	Pes or EAdi	Comments
Gholami et al. [31]	Premature and delayed cycling	11 Patients with pressure controlled-volume guaranteed mode (1204 breaths)	Delta waveform (difference pressure and flow)	Random forest	*Premature cycling:* Sensitivity: 0.89 Specificity: 0.99 *Delayed cycling:* Sensitivity: 0.94 Specificity: 0.98	10-Fold cross validation	Manual annotation by 5 experts of flow, pressure and volume waveforms	No Pes or EAdi available	No patient diagnosis available
Rehm et al. [58] (continues on work from Adams et al. [59])	Double trigger, breath stacking	35 ICU patients (9719 breaths)	16 Features derived from ventilator data (pressure, flow, volume, respiratory rate, I:E ratio)	Supervised ensemble machine learning classifiers (RF, ERTC, GBC, MLP)	*Double trigger:* Accuracy: 0.97 Sensitivity: 0.96 Specificity: 0.98 *Breath stacking:* Accuracy: 0.98 Sensitivity: 0.95 Specificity: 0.99	35-Fold leave-one-subject-out cross validation	Heuristic rules + clinician visual inspection of Paw, volume and flow waveforms (2 experts)	No Pes or EAdi available	Neutralized class imbalances between PVA and non-PVA breaths
Sottile et al. [60]	Double trigger, flow starvation, premature cycling, ineffective effort	62 (At risk for) ARDS patients (2500 breaths)	Manually selected features from flow, pressure and volume waveforms	4 Binary classifiers, one for each PVA (3 models tested for each PVA: Gaussian naive bayes, Adaboost and random forest)	*Cross-validation:* ROC ≥ 0.89 *Separate validation:* Accuracy ≥ 0.54 Sensitivity ≥ 0.23 Specificity ≥ 0.47 ROC ≥ 0.61	62 Patients (2500 breaths for five-fold cross-validation, 500 breaths for separate validation set)	Pressure, flow and volume waveforms mostly reviewed by single expert	No Pes or EAdi available	Premature cycling had the worst results, skewing the outcome metrics
Casagrande et al. [61]	Ineffective effort	8 Patients (500 breaths)	Flow-pressure loops	Linear logistic regression	Accuracy: 0.98 Sensitivity: 0.90 Specificity: 1.0	Tested on same 8 patients (1000 unique breaths)	Manual annotation of pressure, flow and EAdi curves by 3 experts	EAdi available, no Pes available	EAdi was used for training the algorithm, but not for testing
Pham et al. [56]	Reverse trigger	12 Tracings of patients with moderate or severe hypoxemic respiratory failure	Paw and flow waveforms	Combination of methods, including logistic regression and 2nd derivatives	Accuracy: 0.96 Sensitivity: 0.83 Specificity: 0.99	20 Patients that were not used for training (4509 breaths)	Manual annotation of Paw, flow and Pes waveforms by 2 researchers	Pes available, no EAdi available	Pressure control, volume control and pressure support ventilation assessed
Wang et al. [62]	Auto trigger, double trigger, Ineffective effort	44 Patients on NIV	Randomness measures derived from mask pressure, flow, and thoracic and abdominal movement	LightGBM classifier	F-2 score ≥ 0.9 in patients with moderate to high rate of PVA occurrence	Sequential leave-on-out testing	Manual annotation of mask pressure, flow, and thoracic and abdominal movement	Pes or EAdi not available	NIV-data Less generalizable, since performance is dependent on PVA occurrence
Telias et al. [63]	Reverse trigger	36 Patients (± 14,400 breaths)	Paw, flow and Pes waveforms	Decision tree, combined with 1st and 2nd derivatives	Accuracy: 0.95	Same 36 testing patients, different breaths (± 7600 breaths)	Manual annotation of Paw, flow and Pes waveforms by minimally 2 experts	Pes available, no EAdi available	Authors present a method for (quantitative) effort detection as well
Chen et al. [21]	Flow starvation, ineffective effort, reverse trigger, auto trigger, double trigger, premature cycling, delayed cycling	No training data needed; regression was fitted per breath	Hysteresis loop (PV loops)	Piecewise regression models and rule-based thresholds	Accuracy ≥ 0.99 Sensitivity ≥ 0.90 Specificity ≥ 0.97	11 Patients (5500 breaths)	Manual annotation using Paw, flow and PV loops	No Pes or EAdi available	The proposed method that uses PV loops could be integrated in future machine learning algorithms

*ARDS* Acute Respiratory Distress Syndrome, *EAdi *Electrical Activity of the Diaphragm, *ERTC* Extremely Randomized Trees Classifier, *GBC* Gradient Boosted Classifier, *ICU* Intensive Care Unit, *MLP* Multilayer Perceptron, *NIV* Noninvasive Ventilation, *Paw* Airway Pressure, *Pes* Esophageal Pressure, *PVA* Patient-Ventilator Asynchrony, *PV loops* Pressure-Volume loops, *RF* Random Forest, *ROC* Receiver Operating Characteristic

The added value of ML becomes more apparent when the dataset becomes more complex. For instance, Chen et al. [21] attempted to detect 7 different PVAs (flow starvation, ineffective effort, reverse trigger, auto trigger, double trigger, premature cycling and delayed cycling) with a piecewise regression model in combination with rule-based thresholds, using only PV loops obtained from different modes of mechanical ventilation. The performance was tested against clinician waveform review (pressure, flow and PV loops). They reported an accuracy ≥ 0.99, sensitivity ≥ 0.90 and specificity ≥ 0.99 for the detection of individual PVAs, with an average of 0.99, 0.94 and 1.0 for these metrics, respectively. Although the ground truth was not based on measures of patient effort (i.e. Pes or EAdi), these results illustrate the ability of ML to detect multiple PVAs from ventilator data.

## Deep learning (DL)

DL is a sub-field of ML, characterized by the use of neural networks that mimic the structure and function of biological neural networks [26]. It is less transparent than ML and automatically selects features from the raw data. Training of the algorithm can be either supervised or unsupervised and occurs with or without labelled input data, respectively [32]. DL is often seen as a ‘black box’ system, in which the decision-making process is not transparent due to the large number of abstract calculations in the model. However, it can discover otherwise unnoticed, abstract coherences in data and can therefore aid in the complex classification process of PVAs. Studies on such DL models are summarized in Table 3.

**Table 3:** Characteristics of selected deep learning algorithms (studies are arranged chronologically)

Author	Type of PVA	Population	Input data	Algorithm	Performance	Validation	Reference	Pes or EAdi	Comments
Zhang et al. [33]	Double trigger, ineffective effort	Trained on 17 patients (185,385 breaths), validated on 142 patients (330,825 breaths), and vice versa	Pressure and flow waveforms	Deep learning method (RNN model)	*Double trigger:* Accuracy ≥ 0.95 Sensitivity ≥ 0.91 Specificity ≥ 0.96 *Ineffective effort:* Accuracy ≥ 0.92 Sensitivity ≥ 0.86 Specificity ≥ 0.95	Trained on 17 patients (185,385 breaths), validated on 142 patients (330,825 breaths), and vice versa	Manual annotation of pressure and flow waveforms	No Pes or EAdi available	Separate model for double trigger and ineffective effort. They compared their DL model to a ML and RBA model and validated on separate data. Short pre-processing times for the models (max 6 ms)
Pan et al. [64]	Double trigger, ineffective effort, premature cycling, delayed cycling	20 Patients (289.229 breaths)	Flow and pressure waveforms	CNN	Accuracy ≥ 0.97 Sensitivity ≥ 0.98 Specificity ≥ 0.95	Tenfold cross validation (70% of data used for training, 30% for validation)	Manually annotated flow and pressure waveforms	No Pes or EAdi available	Binary classifier for each PVA for each ventilator mode (pressure-controlled and pressure support). Short pre-processing times for the models and low dimensionality allow for real time detection
Baedorf-Kassis et al. [29]	Reverse trigger	15 ARDS patients (23,321 breaths)	Flow, pressure, volume and Pes waveforms, Campbell diagrams (PV loops)	Rule-based software and Modified ResNet network	*RBA:* F1-score: 0.78 Sensitivity: 0.75 Specificity: 0.97 *Deep NN:* F1-score: 0.77 Sensitivity: 0.73 Specificity: 0.97	13 Patients (9923 breaths)	Manually annotated waveforms (flow, pressure, volume) and Pes by 2 experts	Pes available, no EAdi available	NN and an iteration of the RBA used Pes in classification
Bakkes et al. [34]	Delayed inspiration, premature cycling, delayed cycling, ineffective effort	Clinical data: 15 patients on pressure support mode (4275 breaths) Simulated data: 58,876 breaths	Pressure, flow and volume waveforms	Neural network (modified U-net)	Sensitivity ≥ 0.94 PPV ≥ 0.94	4 Approaches: 1) cross validation on clinical data, 2) training on clinical data, testing on simulated data, 3) training on simulated data, testing on clinical data, 4) cross validation on clinical data, but training supplemented with simulated data	Manual annotation of Paw, flow volume and Pes curves by 1 clinician	Pes available, no EAdi available	Reference makes use of only 1 clinician, model evaluated and trained on different combinations of the datasets
De Haro et al. [65]	Flow starvation	28 Patients with square-flow assisted ventilation (6428 breaths)	Paw waveforms	Supervised deep learning networks (RNN and CNN)	*RNN:* Accuracy: 0.88 *CNN:* Accuracy: 0.87	15-Fold holdout cross-validation (80–20 train-validation split)	Manual annotation of Paw and flow curves by 5 experts. Pes was available in a subset of the data	Pes partially available, no EAdi available	Tested a CNN and RNN
Van de Kamp et al. [39]	Premature triggering, delayed triggering, premature cycling, delayed cycling, auto trigger, ineffective effort	15 Patients (7582 breaths)	Pressure, volume and flow waveforms	RNN	Sensitivity ≥ 0.44 PPV ≥ 0.62 F1-score ≥ 0.51 Premature triggering not included due to only 1 occurrence in dataset	15-Fold cross validation	Manually annotated flow, pressure and Pes waveforms	Pes available, no EAdi available	Low latency for labelling data, realtime implementation possible

*ARDS* Acute Respiratory Distress Syndrome, *CNN* Convolutional Neural Network, *DL* Deep Learning, *EAdi* Electrical Activity of the Diaphragm, *ML* Machine Learning, *NN* Neural Network, *Paw* Airway Pressure, *Pes* Esophageal Pressure, *PPV* Positive Predictive Value, *PV loops* Pressure-Volume Loops, *PVA* Patient-Ventilator Asynchrony, *RBA* Rule-Based Algorithm, *RNN* Recurrent Neural Network

Zhang et al. [33] illustrated the added benefits of DL for the detection of double triggering and ineffective efforts, when compared to ML and rule-based algorithms. They showed that while the rule-based algorithm was highly dependent on manually chosen thresholds, ML and DL networks had a more robust performance. Moreover, when validated on different data, DL outperformed ML, especially for the detection of ineffective efforts (minimal accuracy difference of 0.21 (0.92 vs 0.71, respectively)). Lastly, the DL networks had a good extrapolating ability compared to the ML algorithms, demonstrated by less reduction in F1-score (0.98 to 0.89 for DL vs 0.90 to 0.50 for ML). Although the authors showed benefits of DL over ML and rule-based algorithms, an important limitation was the reference dataset, which was annotated without Pes or EAdi available. This limitation was not present in the study of Bakkes et al. [34], where the capabilities of a modified U-net neural network for the detection of delayed inspiration, premature cycling, delayed cycling and ineffective effort was studied. They used a combination of a clinical and simulated dataset, which included Pes waveforms for expert labelling. For the detection of PVAs, they found a minimal sensitivity and positive predictive value (PPV) of 0.94. Furthermore, they showed that artificially generated data can accurately simulate clinical data and therefore aid in the optimization of AI algorithms.

## Licensed software

We define licensed software as software (either rule-based, ML or DL) that is already being used in a commercial product, or as software that is not fully disclosed (likely because it will be commercially deployed). There are several licensed software packages reported for the automated detection of PVAs (Table 4) which could facilitate bedside implementation. An early example is the Better Care® (Better Care, Spain) software, developed by Blanch et al. [35], which focusses on detecting ineffective efforts during expiration using flow curves. The algorithm is rule-based and looks at the deviation of the flow curve compared to a standardized flow curve. When it deviates more than a manually set threshold (> 42%), the system identifies the breath as ineffective effort. When validated on an external dataset with EAdi available, they found a sensitivity and specificity of 0.65 and 0.97, respectively. However, it must be noted that for the ground truth, ineffective efforts were defined as EAdi peaks of > 1 µV above basal EAdi during expiration, not followed by a ventilator breath. This threshold has been shown to be sensitive to electrical and mechanical artefacts, possibly leading to false positive results [22]. A higher threshold might be an appropriate solution to minimize the influence of artefacts, while still retaining sensitivity [36].

**Table 4:** Characteristics of selected licensed software (studies are arranged chronologically)

Author	Type of PVA	Population	Input data	Algorithm	Performance	Validation	Reference	Pes or EAdi	Comments
Blanch et al. [35]	Ineffective effort	8 Patients (1024 breaths)	Paw and flow waveforms	Better Care	*Test (expert opinion):* Sensitivity: 0.92 Specificity: 0.92 *Validation (EAdi):* Sensitivity: 0.65 Specificity: 0.97	Externally validated on 8 patients (9600 breaths)	Manual annotation of Paw and flow waveforms. For validation, EAdi was used (based on a threshold)	EAdi available, no Pes available	Software is (now) integrated with ICU systems. Tested with expert opinion, validated using EAdi from another hospital. Software classifies breaths using expiratory flow waveforms
Phan et al. [37]	Ineffective effort	Algorithm already trained	Delta waveform (difference pressure and flow)	Syncron-E system	Sensitivity: 0.83 Specificity: 0.99	7 Patients (926 breaths), all pressure support ventilated	Manual annotation using Paw, flow, Pes, Pdi and EAdi waveforms (1 clinician)	Pes, Pdi and EAdi available	Compared detection abilities of algorithm vs. clinicians
Chen et al. [38] (continues on work from Su et al. [66])	Flow starvation, double trigger, ineffective effort, premature cycling, delayed cycling, reverse trigger, auto trigger and overshoot	4 Male patients (± 3600 breaths)	Paw, flow and volume waveforms	PVA-RemoteMonitor system	Sensitivity ≥ 0.67 Specificity ≥ 0.90 PPV ≥ 0.74 NPV ≥ 0.99	Same 4 male patients, but different breaths (± 900 breaths)	Manual annotation of flow, volume and pressure waveforms	No Pes or EAdi available	No double triggers or ineffective efforts annotated/present so not validated for those PVAs

*EAdi* Electrical Activity of the Diaphragm, *ICU* Intensive Care Unit, *NPV* Negative Predictive Value, *Paw* Airway Pressure, *Pdi* Transdiaphragmatic Pressure, *Pes* Esophageal Pressure, *PPV* Positive Predictive Value, *PVA* Patient-Ventilator Asynchrony

A more recent algorithm is the Syncron-E^™^ system (Autonomous Healthcare, USA). This software attempts to detect ineffective triggers and uses a random forest classifier based on the delta waveform, as described by Gholami et al. [31]. When Phan et al. [37] validated the software, they found a sensitivity and specificity of 0.83 and 0.99, respectively. The sensitivity for ineffective effort detection was lower than the sensitivity for premature or delayed cycling PVAs as found by Gholami et al. [31], but the specificity remained similar [37]. The difference in sensitivity could be explained by the use of a validation set with Pes and EAdi, or because of the different types of PVAs that were assessed.

Lastly, a recent publication by Chen et al. [38] showed the application of the Remote-VentilateView platform for the detection of eight types of PVA (flow starvation, double trigger, ineffective effort, premature cycling, delayed cycling, reverse trigger, auto trigger and overshoot) in 14 hospitals. No double trigger or ineffective efforts were annotated or present in their data, causing these PVAs to not be validated. Although not licensed at the time of study, the exact underlying algorithm was not disclosed. However, they describe it as a ML algorithm which uses a form of nearest-neighbor classifying and report a sensitivity ≥ 0.67 and specificity ≥ 0.90, with an average of 0.82 and 0.98 for these metrics, respectively [38]. This software seems promising due to its feasibility of implementation in multiple ICUs and its ability to detect multiple types of PVAs with good performance.

## Current challenges

Although results of the abovementioned studies are promising for future bedside AI-assisted PVA detection, we identified several challenges and limitations to be addressed prior to clinical implementation.

*PVA types and definitions:* most of the algorithms studied in this review can only detect a few PVAs, while many forms of PVA exist [15]. To further complicate the reproducibility of research, the definitions of the PVAs are not standardized [25, 39], which warrants consensus of experts in the field.

*Comparison of models:* we clustered the reported studies into four main groups, but the underlying model architectures can differ substantially even within these groups. In addition, mathematical methods are not always fully reported. Both factors hinder consistent classification and comparison between models.

*(Training) data quality:* another limitation is the lack of vast amounts of high-quality data and long recordings. Manual labelling of breathing cycles by clinicians is highly time-consuming and challenging even for expert observers, although the availability of Pes and EAdi as reference for breathing effort can improve labelling accuracy [3, 20]. Ideally, multiple experts should label the breaths to minimize bias and improve labelling accuracy. Inter-observer agreement should be evaluated and reported prior to model development and validation, to assess ground truth labelling quality. ML and DL methods are highly dependent on the training data and models can therefore be biased, emphasizing the need for external validation with high quality data [40, 41].

*Generalizability:* as PVA occurrence could depend on the lung mechanics [42], algorithm development could benefit from training/validation on a wide variety of lung mechanics instead of being trained on a highly selected patient group. This can increase generalizability and enhance performance in new data.

*Reference for effort:* Pes and EAdi are surrogates of patient effort, but mainly reflect inspiratory effort [43]. Expiratory muscles are also frequently engaged in the generation of breathing effort in critically ill patients [44]. Since expiratory muscle effort can cause PVAs [16, 45], PVA occurrence may be underestimated when solely using Pes or EAdi as reference for patient effort. In addition, no data exist on the performance of PVA detection algorithms when using Pes versus EAdi in the labeling process. However, using EAdi as a reference for patient effort might overestimate ineffective effort prevalence, as small-amplitude EAdi peaks can sometimes be the result of cardiac artefacts and insufficient signal filtering [22].

*Towards real-time analysis:* (near) real-time analysis of ventilator data is often lacking. While some automated methods can achieve real-time classification, most reported models included offline or retrospective analyses and were not tested in prospective clinical studies. Although computational processing units and networks have seen vast improvements over the past years in terms of speed, it remains challenging to capture waveform data and integrate real-time application of ML and DL structures on such large amounts of data.

## Towards bedside implementation

**Fig. 1:**
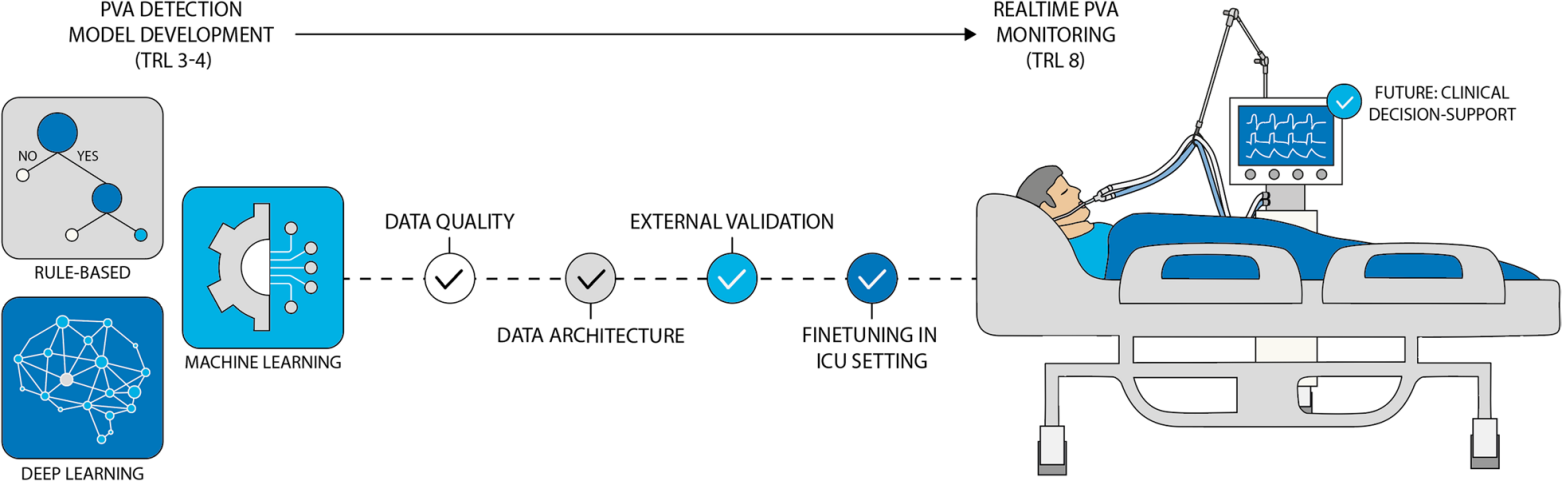
Steps to achieve bench to bedside implementation for Patient-Ventilator Asynchrony (PVA) detection algorithms. *ICU* intensive care unit, *TRL* technology readiness level

Once PVAs can be classified in real-time, their physiological and clinical impact can be further investigated. Several steps could facilitate bedside implementation of AI-based PVA detection. Currently, most algorithms are in the development or validation phase (84%, i.e., 16/19 of the reviewed studies; up to level of readiness 4 or 5, respectively, see Supplemental File 2), while we should finally aim for their use and evaluation in clinical practice (level of readiness 8) [46]. Here, we present a roadmap for the progressing of automated PVA detection algorithms towards implementation in the ICU (see Fig. 1).

High-quality and real-time input data collection is one of the most important factors for AI implementation. Although the ICU environment is characterized by intense data monitoring, the data itself has historically been underused [47–49] and is prone to measurement errors, such as outliers and missing data [46, 50]. ICU data integration therefore remains the main challenge in developing effective tools for PVA analysis [2]. For this to be successful, we emphasize the importance of data quality and data architecture. Data quality directly influences algorithm performance [51] and can be expressed in terms of data quality dimensions, of which accuracy, completeness, consistency, reliability and timeliness are deemed most important [52]. These dimensions are all influenced by the hospital’s information technology infrastructure, collection of data by ICU clinicians, and interoperability of devices, underlining the need for harmonization of data capturing and the connection with context data from the electronic health record.

When front-end data quality is ensured, a data architecture is needed to define the routes for processing, analyzing and storing. This architecture ultimately determines where data processing, and thus algorithm implementation, takes place, influencing processing times and possibilities. Ideally, ICU data should be stored and processed on a central location (e.g., local, or cloud-based solutions [53]) to allow generalizability and smooth implementation of novel algorithms.

Before PVA detection algorithms can be used in a clinical setting, current algorithms need to be validated and generalized. Due to possible overfitting, which is not always noticed in the test dataset, validation on external, new data, is a cornerstone of reliable implementation [54]. For example, an algorithm might perform well on a training and test dataset but can lose its reliability when validated on an external dataset from a different ICU. Factors as differing populations, equipment and frequency of data collection can play a role in this deterioration of performance [46, 55]. Proper external validation ensures the generalizability of the algorithm, which is needed for widespread implementation. However, this remains challenging due to dataset limitations; the level of agreement between clinicians who label the dataset can vary, influencing the ground truth [56]. Furthermore, most datasets lack Pes or EAdi tracings, impacting the labeling accuracy due to the absence of a reference for breathing effort [37]. This is also relevant for asynchronies that are not a result of patient effort per se (e.g., auto-trigger), since Pes or EAdi could aid in the identification of absent patient effort. When ground truth accuracy is deemed sufficient, algorithms should be able to detect the majority of PVA types, either in a single model, or in concurrent models with fast processing times, to allow for (near) real-time monitoring of clusters of PVA.

Last, models should be tested and integrated prospectively in a real-time ICU setting on mechanically ventilated patients, to eventually allow for clinical evaluation. In daily practice, factors as missing measurements, clinical interventions and artefacts (e.g., brief disconnections, cardiac oscillations, leaks) are common, which might be underrepresented in the training, test and validation data. This can influence the performance of the algorithms and emphasizes the need for a real-time evaluation step. When fine-tuning of the model is deemed necessary, a possible solution could be to train the model on artefacts and unstable signals, enabling the algorithm to distinguish an artefact from a PVA, possibly resulting in more accurate PVA detection in a real-time setting. Another possible solution could be to determine the stability of the signal and provide confidence levels of the classifications, such that clinicians are aware that the model output could be based on low probabilities.

## Future perspective

When the abovementioned aspects are correctly implemented, automated PVA detection can become a readily available monitoring tool that allows accurate, continuous monitoring of mechanically ventilated patients, paving the way for personalized ventilation management. This could take away current limitations of PVA research (short-time spans and underestimation of prevalence), allowing the ICU community to study the pathophysiological impact of PVA during the full course of mechanical ventilation and its (causal) effect on clinical outcomes. As a result, it would allow for the development of recommendations and methods to mitigate the possible adverse effects of PVAs. An example is a Clinical Decision Support System (CDSS) that provides clinicians advise on treatment options that lead to the best predicted outcome [57], for instance by proposing ventilator adjustments or modulation of respiratory drive. AI-based detection of PVA and recommendations on how to resolve them may reduce the workload of clinical personnel. When the added value and safety of such system would be validated, a ventilator that automatically adjusts ventilator settings to resolve PVA is not unthinkable for the future. Last, discussions about the ethical and legal considerations surrounding actionable, unsupervised AI in healthcare should take place prior to implementation.

## Conclusion

PVA is a common phenomenon in mechanically ventilated patients and has been associated with adverse events. It is currently identified via visual inspection of ventilator waveforms, but this is highly time-consuming and prone to errors. Automated AI detection methods using rule-based, ML or DL have shown promising results, yet mostly remain in the development or validation stage. For widespread implementation to take place, several steps, including external validation and (near) real-time employment, should be considered. This could finally aid in accurately detecting and mitigating PVAs, reducing clinician’s workload and optimizing personalized mechanical ventilation, and eventually improving clinical outcomes.

## Supplementary Information

Supplementary material 1.

Supplementary material 2.

## Data Availability

Not applicable.

## Abbreviations

AI: Artificial Intelligence
ARDS: Acute Respiratory Distress Syndrome
AUC: Area Under the Curve
CDSS: Clinical Decision Support System
CNN: Convolutional Neural Network
DL: Deep Learning
EAdi: Electrical Activity of the Diaphragm
ERTC: Extremely Randomized Trees Classifier
GBC: Gradient Boosted Classifier
ICU: Intensive Care Unit
ML: Machine Learning
MLP: Multilayer Perceptron
NIV: Noninvasive Ventilation
NN: Neural Network
NPV: Negative Predictive Value
Paw: Airway Pressure
Pdi: Transdiaphragmatic Pressure
Pes: Esophageal Pressure
PPV: Positive Predictive Value
PVA: Patient-Ventilator Asynchrony
PV loop: Pressure-Volume loop
RBA: Rule-Based Algorithm
RF: Random Forest
RNN: Recurrent Neural Network
ROC: Receiver Operating Characteristic
