# Supplementary material for: Let’s get in sync: current standing and future of AI-based detection of patient-ventilator asynchrony
Source: Intensive Care Med Exp. 2025 Mar 21;13:39. doi: 10.1186/s40635-025-00746-8 (PMC11928342; doi:10.1186/s40635-025-00746-8)
Supplement: Supplementary file 1 — Supplementary material 1. [file 40635_2025_746_MOESM1_ESM.docx]

**Supplemental file 1: Confusion matrix metrics**

In this review, we present multiple performance metrics. While some are more known than others, all metrics are based on a 2x2 confusion matrix (see Supplemental Table 1).

Abbreviations: TP = true positives, TN = true negatives, FP = false positives, FN = false negatives.

**Accuracy**

Accuracy is defined as the fraction of correctly predicted conditions, i.e. $\frac{TP+TN}{TP+TN+FP+FN}$

**Sensitivity**

Sensitivity (also known as recall) is defined as the proportion of positive condition cases that are correctly identified, i.e. $\frac{TP}{TP+FN}$

**Specificity**

Specificity is defined as the probability that cases with a negative prediction do not actually have the condition, i.e. $\frac{TN}{TN+FP}$

**Positive Predictive Value (PPV)**

Positive predictive value (also known as precision) is defined as the probability that cases with a positive prediction actually have the condition, i.e. $\frac{TP}{TP+FP}$

**Negative Predictive Value (NPV)**

Negative predictive value is defined as the probability that cases with a negative prediction indeed do not have the condition, i.e. $\frac{TN}{TN+FN}$

**F1-score**

F1-score is a more abstract measure of predictive performance. It is calculated using sensitivity and PPV, and is a balanced mean of the two. It is defined as $\frac{2*PPV*Sensitivity}{PPV+Sensitivity}$, which can be rewritten as $\frac{2*TP}{2*TP+FN+FP}$

**F2-score**

F2-score is a variant of the F1-score, but places more emphasis on sensitivity than PPV. In other words, it places more emphasis on the detection of a condition than on the reliability of the positive prediction. It is defined as $\frac{5*PPV*Sensitivity}{4*PPV+Sensitivity}$, which can be rewritten as $\frac{5*TP}{5*TP+4*FN+FP}$

**Receiver Operating Characteristic curve (ROC-curve)**

A Receiver operating characteristic (ROC) curve is a graphical representation that can be used to evaluate the performance of a classification model. The curve shows the trade-off between the sensitivity and specificity at various thresholds. It has the sensitivity on the y-axis, while 1-specificity is on the x-axis. The top left corner therefore represents an ideal classifier, with 100% sensitivity and 100% specificity. The diagonal from the bottom left to top right corner is the line that represents a random classifier; the classification of a random classifier can be seen as 50/50 or a ‘flip of a coin’. Classifiers that are closer to the top left are thus better performing classifiers, while models that are beneath the diagonal perform worse than random classification. An example of ROC-curves is given in Supplemental Figure 1.

*Supplemental table 1: Confusion matrix*

**Actual condition**

|  | **Predicted condition** | |
| --- | --- | --- |
| Total population | Predicted Positive | Predicted Negative |
| Positive | True Positive (TP) | False Negative (FN) |
| Negative | False Positive (FP) | True Negative (TN) |


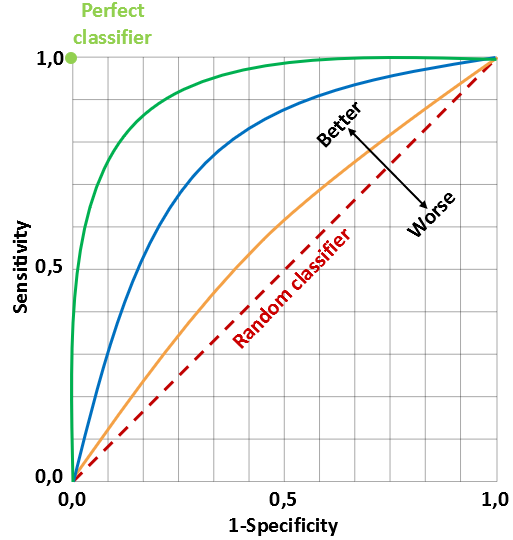


*Supplemental Figure 1: Example of ROC-curves of 4 different (theoretical) classifiers, including a random classifier reflecting a fifty-percent chance guess.*
