# Supplementary material for: Let’s get in sync: current standing and future of AI-based detection of patient-ventilator asynchrony
Source: Intensive Care Med Exp. 2025 Mar 21;13:39. doi: 10.1186/s40635-025-00746-8 (PMC11928342; doi:10.1186/s40635-025-00746-8)
Supplement: Supplementary file 2 — Supplementary material 2. [file 40635_2025_746_MOESM2_ESM.pdf]

Supplemental File 2: Technology Readiness Levels

Technology Readiness Levels (TRLs) are initially developed by NASA to systematically assess the maturity of a technology [1]. The TRLs have been adapted by the European Union, among others, and are often used in the context of funding [2, 3]. TRLs range from 1-9 and respectively reflect the range from observing a basic principle to an actual proved system in an operational environment. The TRL levels and their corresponding definitions can be found in Supplemental table 2.

Supplemental table 2: Technology Readiness Levels (TRLs), according to their EU definition.

TRL	Definition
**1**	Basic principles observed
**2**	Technology concept formulated
**3**	Experimental proof of concept
**4**	Technology validated in lab
**5**	Technology validated in relevant environment (industrially relevant environment in the case of key enabling technologies)
**6**	Technology demonstrated in relevant environment (industrially relevant environment in the case of key enabling technologies)
**7**	System prototype demonstration in operational environment
**8**	System complete and qualified
**9**	Actual system proven in operational environment (competitive manufacturing in the case of key enabling technologies; or in space)

Supplemental References:

1. Mankins J (1995) Technology Readiness Level – A White Paper

2. Héder M (2017) From NASA to EU: the evolution of the TRL scale in Public Sector Innovation. The Innovation Journal 22:1

3. Bruno I, Lobo G, Covino BV, et al (2020) Technology readiness revisited: a proposal for extending the scope of impact assessment of European public services. In: Proceedings of the 13th International Conference on Theory and Practice of Electronic Governance. ACM, Athens Greece, pp 369–380
